# Aerosolized nanoliposomal carrier of remdesivir: an effective alternative for COVID-19 treatment *in vitro*

**DOI:** 10.2217/nnm-2020-0475

**Published:** 2021-05-13

**Authors:** Richa Vartak, Suyash M Patil, Aishwarya Saraswat, Manali Patki, Nitesh K Kunda, Ketan Patel

**Affiliations:** ^1^College of Pharmacy & Health Sciences, St. John's University, New York, NY 11439, USA

**Keywords:** COVID-19, drug delivery, liposomes, localized treatment, nebulizer, pulmonary, remdesivir

## Abstract

**Aim:** To formulate an aerosolized nanoliposomal carrier for remdesivir (AL-Rem) against coronavirus disease 2019. **Methods:** AL-Rem was prepared using modified hydration technique. Cytotoxicity in lung adenocarcinoma cells, stability and aerodynamic characteristics of developed liposomes were evaluated. **Results:** AL-Rem showed high encapsulation efficiency of 99.79%, with hydrodynamic diameter of 71.46 ± 1.35 nm and surface charge of -32 mV. AL-Rem demonstrated minimal cytotoxicity in A549 cells and retained monolayer integrity of Calu-3 cells. AL-Rem showed sustained release, with complete drug release obtained within 50 h in simulated lung fluid. Long-term stability indicated >90% drug recovery at 4°C. Desirable aerosol performance, with mass median aerodynamic diameter of 4.56 ± 0.55 and fine particle fraction of 74.40 ± 2.96%, confirmed successful nebulization of AL-Rem. **Conclusion:** AL-Rem represents an effective alternative for coronavirus disease 2019 treatment.

## Lay abstract

Remdesivir is one of the first drugs approved for the treatment of coronavirus disease 2019. Currently, it is administered via an injection into the bloodstream. This means that the drug circulates around the entire body and only a limited amount reaches the diseased site – the lungs. Frequent dosing is therefore required, which needs expert personnel and multiple hospital visits and can result in serious side effects. In this study, the authors developed specialized, nanosized particles containing the drug remdesivir that can be administered directly into the lungs. This could drastically minimize side effects, enhance efficacy and allow easy self-administration at home. The results of the study are promising but require additional investigation.

## Graphical abstract

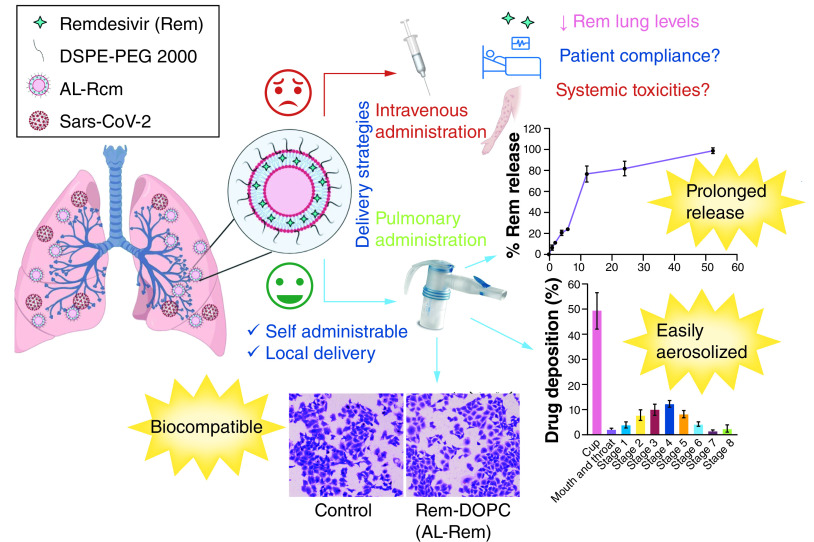

Coronavirus (the Latin *corona* meaning “crown”) was first isolated from a patient's respiratory tract in 1965 [1]. Volunteer inoculation and an epidemiological study confirmed coronaviruses to be associated with respiratory illnesses [2]. SARS coronavirus 2 (SARS-CoV-2) was first detected in Wuhan, China, and has so far affected 50 million people, with 1 million associated deaths [3]. The reproduction number (i.e., R0 value) of SARS-CoV-2 lies between 2.43 and 3.10 [2]. This indicates that the potential for the SARS-CoV-2 virus to infect and transmit among humans is very strong, unlike Middle East respiratory syndrome coronavirus, which has an R0 value of less than 1 [4]. Multiple routes are involved in SARS-CoV-2 transmission, with droplets and aerosols accounting for maximum transmission [5]. The viral load gets entrapped within mucus or saliva globs, and the particle size greatly influences the region in which the particles are deposited. Based on the aerosol diameter, they can either deposit and infect the deep lungs or, if larger, get trapped within the upper airways [5,6].

The main factor governing the severity of infection is the presence of specific receptors, such as ACE2, CD4, CCR5, TMPRSS2, CD81 and JAM, that enable viral attachment and entry. The entry of coronavirus into the pulmonary cells is primarily mediated by the surface-anchored spike protein that binds to the cellular receptor target (ACE2) present on the cell surface [7]. As ACE2 is predominantly found on the mucosal lining of the nose and lungs, it greatly facilitates entry to and infection of the respiratory tract [8]. Following receptor attachment, the virus enters the cell cytoplasm, after which the viral proteins are uncoated, releasing viral genetic material. Coughing and sneezing lead to shedding of viral progenies from the respiratory tract which can be easily encountered in nasal swabs [9].

With the ongoing effort toward developing an ideal, effective vaccine for coronavirus disease 2019 (COVID-19), scientists are repurposing currently available drugs to treat hospitalized patients. Among the thousands of drugs screened for activity against SARS-CoV-2, some of the US FDA-approved drugs include remdesivir (Rem), dexamethasone, hydroxychloroquine and chloroquine. Rem has been explored for several indications and has currently been repurposed for COVID-19 treatment [10]. Rem (GS-5734), a potent viral RNA-dependent RNA polymerase inhibitor, was recognized to be a promising drug candidate against SARS-CoV-2 after *in vitro* testing [11]. A randomized controlled trial of Rem in the treatment of SARS-CoV-2 was initiated by a group of researchers in February 2020 [12]. Early results indicated accelerated recovery of hospitalized, severely ill COVID-19 patients. The currently used medicament Veklury, an injectable form of Rem, is prescribed for adults and children older than 12 years who weigh ≥40 kg and are hospitalized for COVID-19. As the viral particles are known to reside and proliferate within the respiratory airways, effective Rem concentration must be attained within the lungs [9]. Certain findings indicate that following intravenous dosing of Rem (100–200 mg), and via interpretation of human and animal data, the concentration of Rem and its active metabolite in the lungs is inadequate to inhibit SARS-CoV-2. This could be due to the physicochemical and pharmacokinetic properties of the drug leading to low tissue distribution and penetration, especially in the lungs; thus, as reported by Sun, to show efficacy, the concentration of Rem required to inhibit SARS-CoV-2 in cell culture would be IC_50_ >7.7 μM and IC_90_ >17.6 μM of the active metabolite [13]. For such a high concentration of Rem to directly reach the lungs, localized pulmonary delivery would be highly advantageous.

Pulmonary delivery would provide high lung concentrations, mitigate systemic exposure and toxicities and reduce dosing frequency [14]. Inhalation via nebulization is one approach that provides easy and convenient drug delivery for patients [15]. Delivering 50 mg Rem for 30 min via nebulizer would be the fastest approach and would deliver the drug directly to the airways. This could aid in healing the infection earlier and lessen the likelihood of prolonged hospitalization. However, the main issue with delivering Rem to the lungs is its poor solubility and aqueous instability. It is also more likely to undergo degradation during storage in an aqueous environment [16]. Nanocarrier-based pulmonary drug delivery systems like liposomes possess unique properties, including small size (∼100 nm) and circumvention of first-pass effect, making them suitable drug carriers for enhanced pulmonary deposition. Moreover, the large surface area of the lungs and the high permeability of the pulmonary epithelium offer unique pulmonary targeting options. Unlike parenteral delivery, the inhalation route for controlled release systems like liposomes can result in localized drug action in the lungs for prolonged periods, improving the therapeutic outcome of the medication, increasing drug aqueous stability and reducing systemic adverse effects [17].

Therefore, the current study aimed to formulate a stable aerosolized nanoliposomal carrier for Rem (AL-Rem) that would be biocompatible, efficient in drug delivery and able to successfully fulfill the criteria associated with nebulization. The liposomal solution would be directly nebulized to the pulmonary region as an improved and localized therapy against COVID-19.

## Methods

### Materials

Rem was purchased from Chemietek (IN, USA). DPPC and PE 18:0/18:0-PEG2000 were obtained from Lipoid (Ludwigshafen, Germany). Cholesterol, chloroform, NMP, tetrahydrofuran and TFA were acquired from Sigma-Aldrich (MO, USA). SBE-β-CD was kindly donated by Ligand Pharmaceuticals (CA, USA). DOPC and lactate dehydrogenase (LDH) assay kit were obtained from Cayman Chemical Company (MI, USA). Transwell^®^ insert plates were procured from VWR International (PA, USA). ACN, methanol, acetone and HPLC-grade water were purchased from Thermo Fisher Scientific (MA, USA).

### HPLC analysis

Rem was chromatographically separated using a Waters Alliance^®^ HPLC system with a 2998 photodiode array detector (NJ, USA). A 250 × 4.6-mm, 5-μm Hypersil ODS^®^ column (Thermo Fisher Scientific) was used as the reverse phase C18 column maintained at 25°C. An optimum mobile phase ratio of 65:35 (ACN with 0.1% TFA:phosphate buffer with pH adjusted to 3.5) was used with a single injection volume of 10 μl and a flow rate of 1 ml/min. Autosampler assembly was used to load the samples from the carousel, and eluted drug peak signal was detected at 247 nm. Data were analyzed using Empower 3 software (Waters).

### Stability study of Rem

Currently, there are no reports on the stability of Rem. Therefore, it was necessary to investigate pH, thermal stability and photostability of Rem. To determine stability over a pH range, Rem stock (1 mg/ml) was prepared by solubilizing in NMP. The stock solution was diluted with a 1:1 (v/v) mixture of ACN:0.1N hydrochloric acid with a pH of 1.2, ACN:phosphate buffer with a pH of 3.5 and ACN:phosphate buffer with a pH of 7.4 to achieve a final concentration of 50 μg/ml [18]. Each batch was analyzed in triplicate and maintained at different temperature conditions (i.e., 4°C, 25°C and 37°C). The samples were collected after 24 h and analyzed using HPLC. The photostability of Rem was examined under UV radiation. Based on the HPLC method for Rem, the solvent mixture chosen for sample preparation was ACN:HPLC water (1:1). The Rem stock prepared in NMP was diluted in a scintillation vial with ACN:HPLC water (1:1) to achieve a final concentration of 50 μg/ml. To determine the photodegradation of Rem, the vial was placed in a UV chamber. At various time intervals of 0, 1, 3 and 5 days, the sample was withdrawn and the drug content analyzed using HPLC.

### Preparation of Rem liposomes

The solubility of Rem in different organic solvents (i.e., acetone, methanol, THF and ACN) was tested prior to formulation development. Different commonly used methods for preparing Rem-loaded liposomes were screened. First, an ethanol injection method was used wherein lipids and Rem were dissolved in absolute ethanol and injected into water at 55°C. The resultant dispersion was probe-sonicated at 30% amplitude for 2 min, followed by centrifugation to separate free Rem.

Another method used to prepare Rem-loaded liposomes is the modified hydration method, as described by Patel *et al.* [19]. The compositions of various batches prepared using different concentrations of lipids are given in Table 1. Briefly, Rem and a weighed quantity of lipids were dissolved in a mixture of THF and chloroform. The mixture was subsequently added to and adsorbed on parenteral-grade mannitol (200 μm) dropwise with continuous stirring at 60°C and left overnight to evaporate the organic solvent. The resultant adsorbed powder was dispersed thoroughly in warm water at 55°C, and the formed dispersion was probe-sonicated at 30% amplitude for 2 min. Liposomes were centrifuged at 490 relative centrifugal force (RCF) for 5 min to separate free Rem (if any), and the supernatant was analyzed for its physicochemical characteristics and entrapment efficiency. Final nanoliposomes of Rem (AL-Rem) were optimized as per Table 1.

**Table 1.** Composition of Rem liposomal batches prepared using modified film hydration.

	Liposome batch	Cholesterol (mM)	DSPE-PEG2000 (mM)	DPPC (mM)	DOPC (mM)	Rem (mg)
1.	Lipo-DPPC	26	4	82	–	2.5
2.	Lipo-DODP	26	4	41	38	2.5
3.	Lipo-DODP	26	4	61.5	19	2.5
4.	Lipo-DOPC1	26	4	–	76	2.5
5.	Lipo-DOPC2	26	4	–	76	5

DODP-DOPC and DPPC (1:1).

Rem: Remdesivir.

### Physicochemical characterization of AL-Rem

AL-Rem was characterized based on particle size, polydispersity index and zeta potential using a Zetasizer Nano ZS dynamic light scattering particle size analyzer (Malvern Panalytical, Royston, UK). Samples were accurately diluted (i.e., 150 μl of liposomes in 3 ml of purified water). Diluted samples were loaded into disposable cuvettes and analyzed at 25°C with a scattering angle of 173°. For entrapment efficiency determination, AL-Rem was loaded in Amicon ultra centrifugal filters (50,000) and centrifuged at 1110 RCF for 10 min (Darmstadt, Germany). The decant was analyzed for free drug using HPLC analysis [20]. The encapsulation efficiency of Rem was expressed as the percentage of drug encapsulated and calculated using the following formula:

$$\text{Percent}\;\text{encapsulated}=\frac{\left[(\text{Total}\;\text{Rem})-(\text{Free}\;\text{Rem})\right]}{\left(\text{Total}\;\text{Rem}\right)}\times 100\%$$

### *In vitro* cytotoxicity assays

The cytotoxicity of Rem liposomes prepared using DPPC and DOPC was assessed on A549 cells (American Type Culture Collection, VA, USA) using MTT assay. RPMI medium, supplemented with L-glutamine, 1% sodium pyruvate and 10% fetal bovine serum, was used as growth medium. For this assay, 1 × 10^4^ cells/well were seeded in a 96-well plate and allowed to attain confluency overnight under humdified conditions at 37°C and 5% CO_2_. The medium in each well was withdrawn, replaced with the treatment (Rem-loaded DOPC/DPPC liposomes ranging from 2.5 to 0.08 mg/ml) and incubated for 3 h. The treatments were substituted with fresh media and further incubated to analyze any post-treatment toxicities. After 24 h, cell viability was assessed using MTT assay.

Crystal violet assay was performed to examine the morphological changes in the presence of the treatment groups. As per the aforementioned protocol, after 24-h incubation, the cells were washed with phosphate-buffered saline and fixed using 4% v/v glutaraldehyde. Later, the fixed cells were stained with 0.5% w/v crystal violet. Excess crystal violet was washed out using HPLC-grade water, and plates were air-dried overnight. Images were captured at x20 magnification using an inverted microscope (Evos XL core imaging system; Thermo Fisher Scientific).

### Cellular uptake study

To assess qualitative cellular uptake, Coumarin 6 (C6) fluorescent dye-loaded nanoliposomes were prepared following the same protocol mentioned earlier. A549 cells were plated in a 96-well plate at a density of 10,000 cells/well and maintained at 37°C and 5% CO_2_ overnight. The cells were treated with free C6 (i.e., C6 solution) and C6-loaded nanoliposomes at a concentration of 2.5 μg/ml each for a duration of 2 h. Later, the cells were washed with phosphate-buffered saline and further incubated with 1 μg/ml of DAPI nuclear stain for 15 min. Cells were washed with phosphate-buffered saline and images were captured at x20 magnification using an EVOS FL auto cell imaging system (Thermo Fisher Scientific).

### LDH assay

The release of LDH enzyme present in a cell is an indication of damage caused to the plasma membrane. Cells were cultivated in 96-well plates as per the similar protocol mentioned earlier. Cells were treated with the highest concentration of AL-Rem (i.e., 2.5 mg/ml), and an equivalent volume of blank liposomes was tested in other wells. To completely nullify the effect of liposome turbidity on the absorbance values, a blank control comprising only treatment without cells was also evaluated. A direct comparison of the treatment groups was performed with the positive controls (i.e., Triton X-100 and 0.1% w/v SLS). After incubating with treatment for 3 h, the quantity of LDH released or leaked was measured using an LDH assay kit (Cayman Chemical Company). Required reagents were prepared as indicated in the manufacturer's protocol. Well plates were centrifuged and supernatant was withdrawn. Each supernatant with reagent was analyzed at 490 nm for the concentration of LDH released in the presence of each treatment using an Epoch 2 plate reader (BioTek Instruments, VT, USA) [21]. The interference of the liposomal formulation in the absorbance measurement was eliminated by subtracting it from the blank control (treatment without cells). The percentage of LDH activity was calculated using the following formula:

$$\%\;\text{LDH}\;\text{activity}=\frac{[(\text{Experimental}\;\text{value}\;\text{A}490)-(\text{Spontaneous}\;\text{release}\;\text{A}490)]}{\left[(\text{Maximum}\;\text{release}\;\text{A}490)-(\text{Spontaneous}\;\text{release}\;\text{A}490)\right]}\times 100$$

### Epithelial integrity study

The effect of the liposomal formulation on the integrity of airway lung epithelial cells was examined using the Calu-3 cell line. Briefly, 1 × 10^5^ cells were seeded per polycarbonate insert (0.33 cm^2^, 0.4 μm) in 24-well Transwell^®^ plates. The growth medium used was Eagle's Minimum Essential Medium supplemented with 10% fetal bovine serum. Transepithelial electrical resistance (TEER) was measured each day following medium replacement using a Millicell ERS-2 (MilliporeSigma, NH, USA) device probe until a TEER value of 900–1000 ohm-cm^2^ was achieved, indicative of monolayer formation. Two commonly used drug delivery approaches (i.e., liposomes and SBE-β-CD) were screened for their effect on epithelial integrity. Briefly, DOPC-based liposomes (equivalent to 1 mg/ml of AL-Rem) and SBE-β-CD (50 mg/ml) were dissolved in the medium. Growth medium in the apical chamber at t = 0 was replaced with the respective treatments and resistance was measured at 0.5, 1, 2, 4, 6 and 24 h. Inserts with medium alone were used as negative control.

### *In vitro* release study

*In vitro* release study was carried out to compare the release profile of Rem from Rem solution and AL-Rem in simulated lung fluid (SLF) conditions. Briefly, 10,000 molecular weight cutoff Slide-A-Lyzer dialysis cassettes (0.1–3 ml; Thermo Fisher Scientific) were hydrated in the prepared SLF medium for 10 min prior to use. As DPPC is an essential lung surfactant, modified SLF-3 medium (i.e., SLF-4) was prepared as previously described in the literature [21]. Briefly, concentrated, blank DPPC liposomes were added to the SLF-3 fluid to account for the lung surfactant. DPPC liposomes were prepared using the thin-film hydration method. A total of 100 mg of DPPC was dissolved in a 1:1 mixture of chloroform:methanol and subjected to rotary evaporation to form a lipid film. The lipid film was hydrated with 100 ml distilled water and agitated at 55°C for 2 h. The dispersion was bath-sonicated for 2 h at 55°C. The solution was mixed with lung fluid and the pH was adjusted to 7.4 [22].

For the release study, hydrated cassettes were loaded with 1 ml of the Rem solution and AL-Rem formulation separately from one port using a 19G1_1/2_ TW BD filter needle attached to a 3-ml syringe. Rem solution was prepared by solubilizing 2.5 mg of Rem in 1 ml of 15% w/v SBE-β-CD solution using the coprecipitation method. The filled cassettes were immersed in a beaker containing 150 ml of SLF-4 medium using a floater, and the assembly was constantly stirred at 250 rpm. Periodic withdrawals at time points 0.5, 1, 2, 4, 6, 12, 24 and 50 h were made and immediately replenished with an equivalent quantity of fresh dissolution medium. The aliquots were accurately diluted with ACN, centrifuged at 16,300 RCF for 5 min and analyzed for drug release from both formulations using HPLC.

### Liposomal stability study

The stability of AL-Rem was analyzed in terms of percentage of drug content, particle size and polydispersity index. Briefly, AL-Rem at 2.5 mg/ml was subjected to different temperature conditions (i.e., 4°C and 25°C). Aliquots of these samples were analyzed for percentage of drug remaining after 2 and 4 weeks using HPLC. Physicochemical characterization of the liposomal formulation was also performed at the end of 4 weeks using a dynamic light scattering particle size analyzer [23].

### *In vitro* pulmonary drug deposition

Pulmonary drug deposition of the AL-Rem formulation was evaluated using a Next Generation Impactor (NGI) 170 (MSP Corporation, MN, USA) as described previously [24]. A stainless steel induction port (i.e., United States Pharmacopeia throat adaptor attachment) and specialized stainless steel NGI^™^ gravimetric insert cups (model 170; MSP Corporation) were attached to the NGI. Prior to use, refrigeration for 90 min at 4°C was performed to cool the equipment, minimizing the heat transfer evaporative effects of the nebulized droplets, which cause droplet shrinkage and impact deposition. The AL-Rem formulation (2 ml) was placed into the PARI LC PLUS^®^ nebulizer cup of a PARI FAST-NEB compressor system (Boehringer Ingelheim, CT, USA) attached to a rubber mouthpiece and linked to the NGI. A flow rate of 15 l/min was delivered using an HCP5 vacuum pump (Copley Scientific, Nottingham, UK) and adjusted using a DFM 2000 flow meter (Copley Scientific). Samples were collected from the different stages using a lysis solvent (a binary mixture of ACN and water), subjected to centrifugation (13,000 RCF) to separate the encapsulated drug and analyzed using HPLC. Mass median aerodynamic diameter (MMAD) and geometric standard deviation, the crucial parameters for aerosol performance, were calculated by computing AL-Rem deposition at each stage of the NGI using log probability analysis (n = 3). The fine particle fraction (FPF) percentage was defined as the fraction of emitted dose (total amount of drug that exited the nebulizer) deposited in the NGI at each stage [14].

### Statistical analysis

Each data set illustrated the mean ± standard deviation. Student's *t*-test and one-way analysis of variance were used to calculate the significant difference values in the treatment groups. The software used for analysis was Prism 5.0 (GraphPad Software, CA, USA), where p < 0.05 between each group was measured to be a statistically significant difference between the evaluated groups.

## Results

### HPLC method

HPLC analysis presented a sharp peak of Rem with a retention time of 4.82 ± 0.5 min within a run time of 8 min. The developed HPLC method was utilized to analyze all the samples from stability, release, physicochemical characterization and NGI analysis.

### Stability study of Rem

**Figure 1.**
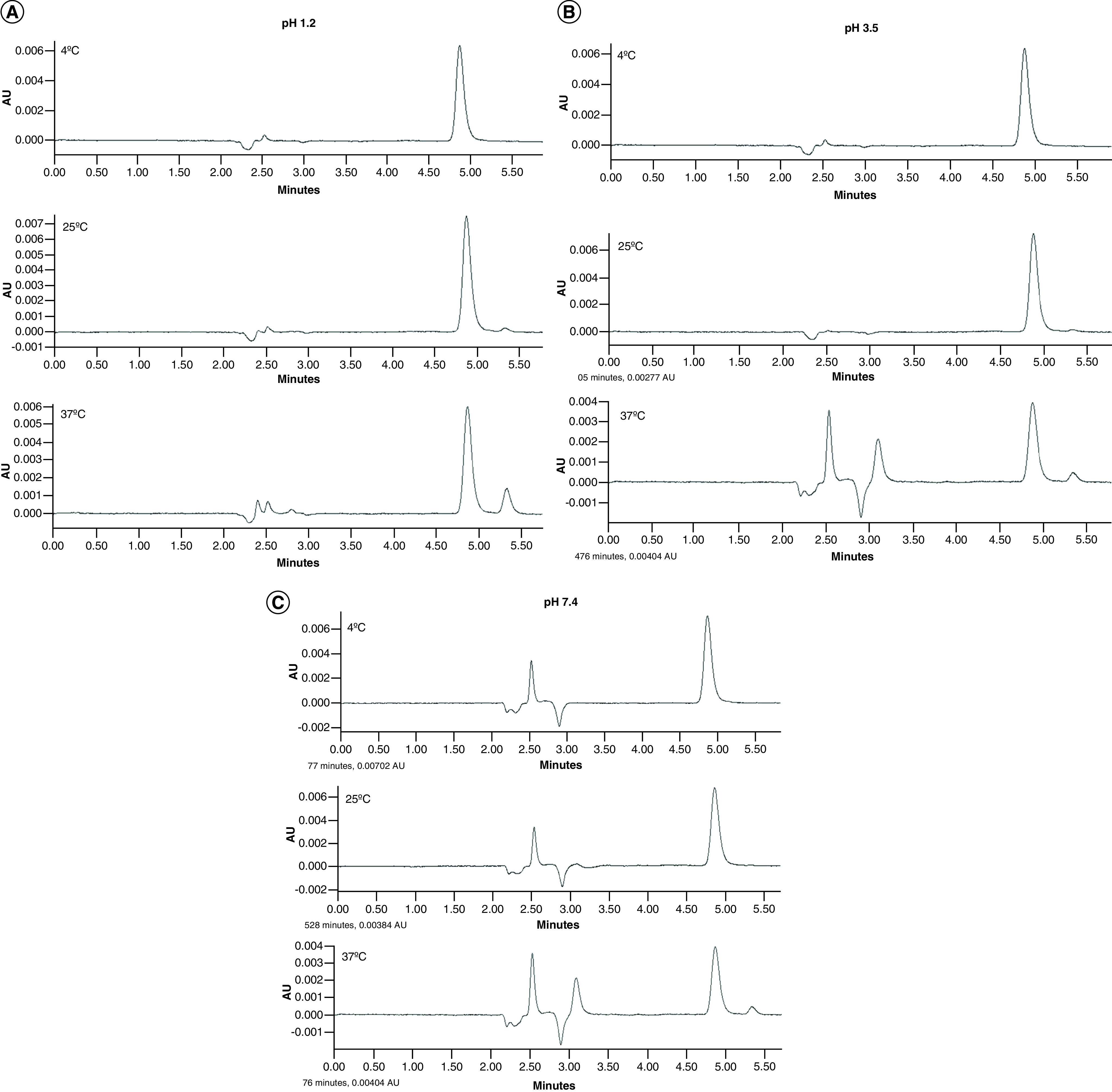
Stability study of remdesivir in different temperature and pH conditions. HPLC chromatograms indicating the stability assessment of Rem at pH **(A)** 1.2, **(B)** 3.5 and **(C)** 7.4 under different temperature conditions. Rem showed significant degradation at 37°C in all pH conditions.

Rem was found to be stable at 4°C in all tested pH conditions (i.e., 1.2, 3.5 and 7.4). However, as shown in Figure 1A, Rem in pH 1.2 stored at 25°C and 37°C revealed a degradation peak at 5.25 min. Moreover, a significant amount of degradation was observed at 37°C in all pH conditions (Figure 1A–C). Substantial degradation of Rem at higher temperature was evident in the reduction in peak height seen at all pH levels. The concentration of Rem after UV exposure for 5 days was nearly the same (i.e., approximately 50 μg/ml).

### Preparation of AL-Rem

Among the commonly used methods, ethanol injection showed rapid precipitation of Rem. Because of the poor liposomal stability, the modified hydration method was adopted. Rem solubility in different organic solvents was evaluated prior to formulation development (data not shown). Organic solvents like THF and methanol showed the highest Rem solubilizing capacity in comparison to acetone and ACN, which showed solubility of <1 mg/ml. Therefore, THF was used to solubilize Rem, with chloroform added to solubilize lipids. The most commonly used phospholipids (i.e., DPPC and DOPC) were evaluated. Batches 1, 2 and 3 (Table 1) prepared using DPPC showed instability and drug precipitation over time. Increasing molar ratio of DPPC in comparison to DOPC showed a decrease in stability. Therefore, a liposomal formulation with DOPC alone was prepared. The Rem-DOPC liposomes (AL-Rem) showed complete drug entrapment with no visible precipitation.

### Physicochemical characterization of Rem liposomes

The optimized Rem-loaded DOPC liposomes that were recognized as AL-Rem showed a hydrodynamic size of 71.46 ± 1.35 nm with a polydispersity index of 0.202 ± 0.085. The zeta potential of the liposomes was found to be –32 ± 2 mV. The encapsulation efficiency of AL-Rem was found to be 99.79%, indicating complete entrapment of Rem lipid bilayers.

### *In vitro* cytotoxicity assay

**Figure 2.**
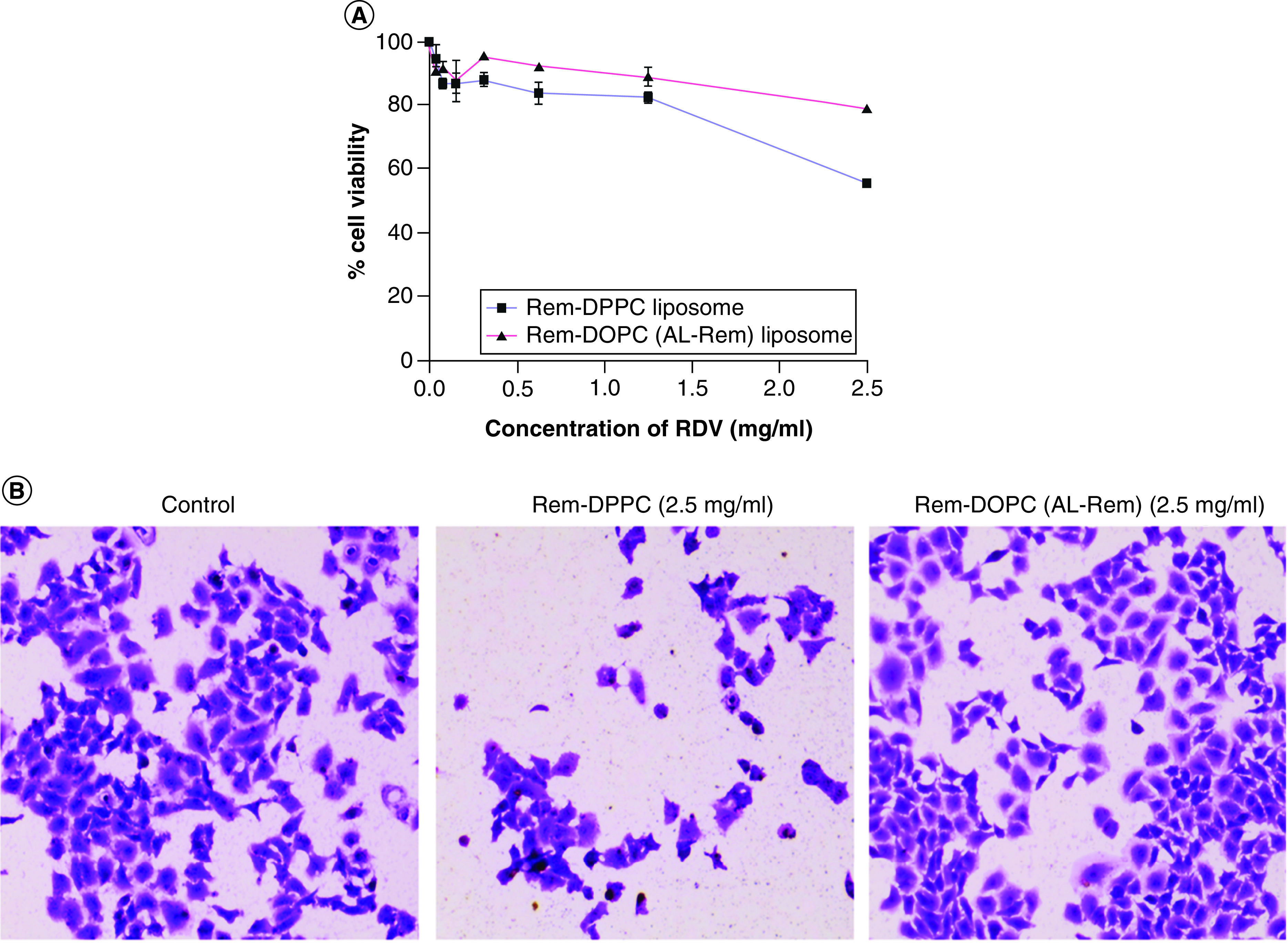
*In vitro* cytotoxicity studies in A549 cell line. **(A)** Cell viability of A549 cells in the presence of Rem-DPPC and Rem-DOPC liposomes. Highest concentration of Rem-DPPC liposomes showed 1.5-fold higher toxicity than Rem-DOPC liposomes. **(B)** Crystal violet staining 24 h after 3-h incubation showed considerable reduction in cell number for Rem-DPPC liposomes. Rem-DOPC liposomes showed confluency similar to control. Each data point represents mean ± SD (n = 3). AL-Rem: Aerosolized nanoliposomal carrier for remdesivir; Rem: Remdesivir; SD: Standard deviation.

As shown in Figure 2A, apart from the physical instability associated with DPPC liposomes, significant toxicity to lung carcinoma cells was seen. By contrast, DOPC-based liposomes (i.e., AL-Rem) were safe and showed cell viability of more than 90% even at the highest concentration of 2.5 mg/ml Rem. DPPC-based liposomes at a similar concentration showed drastic decrease in cell viability to 60%. The results were further confirmed by performing crystal violet staining to study cell morphology. As depicted in Figure 2B, Rem-DPPC liposomes showed a significant decrease in cell number as well as disrupted cell surface compared with Rem-DOPC liposomes (AL-Rem). By contrast, confluency and cell morphology of the group treated with Rem-DOPC liposomes (AL-Rem) were similar to the control.

### Cellular uptake study

**Figure 3.**
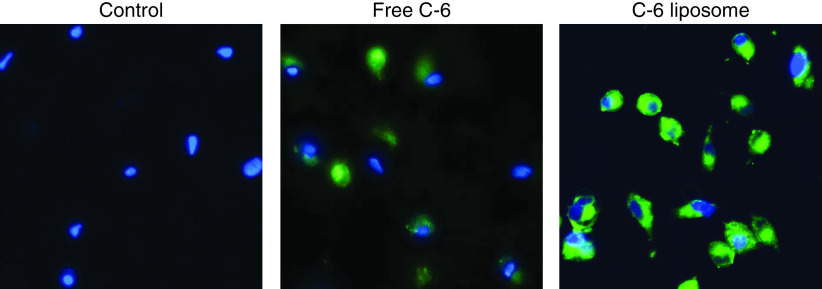
Cellular uptake study of free C6 and C6-loaded liposomes in A549 cells. Higher fluorescence intensity was observed in C6-loaded liposomes compared with free C6 liposomes. Images were captured under x20 magnification.

A qualitative cellular uptake assay was carried out in A549 (i.e., lung carcinoma) cells. For this experiment, liposomes were formulated by replacing Rem with C6 as the active ingredient to allow better visualization of liposomal uptake and accumulation. As per Figure 3, fluorescent images revealed a higher green fluorescence intensity in C6-loaded nanoliposomes in comparison to free C6 liposomes treated at the same concentration.

### LDH assay

**Figure 4.**
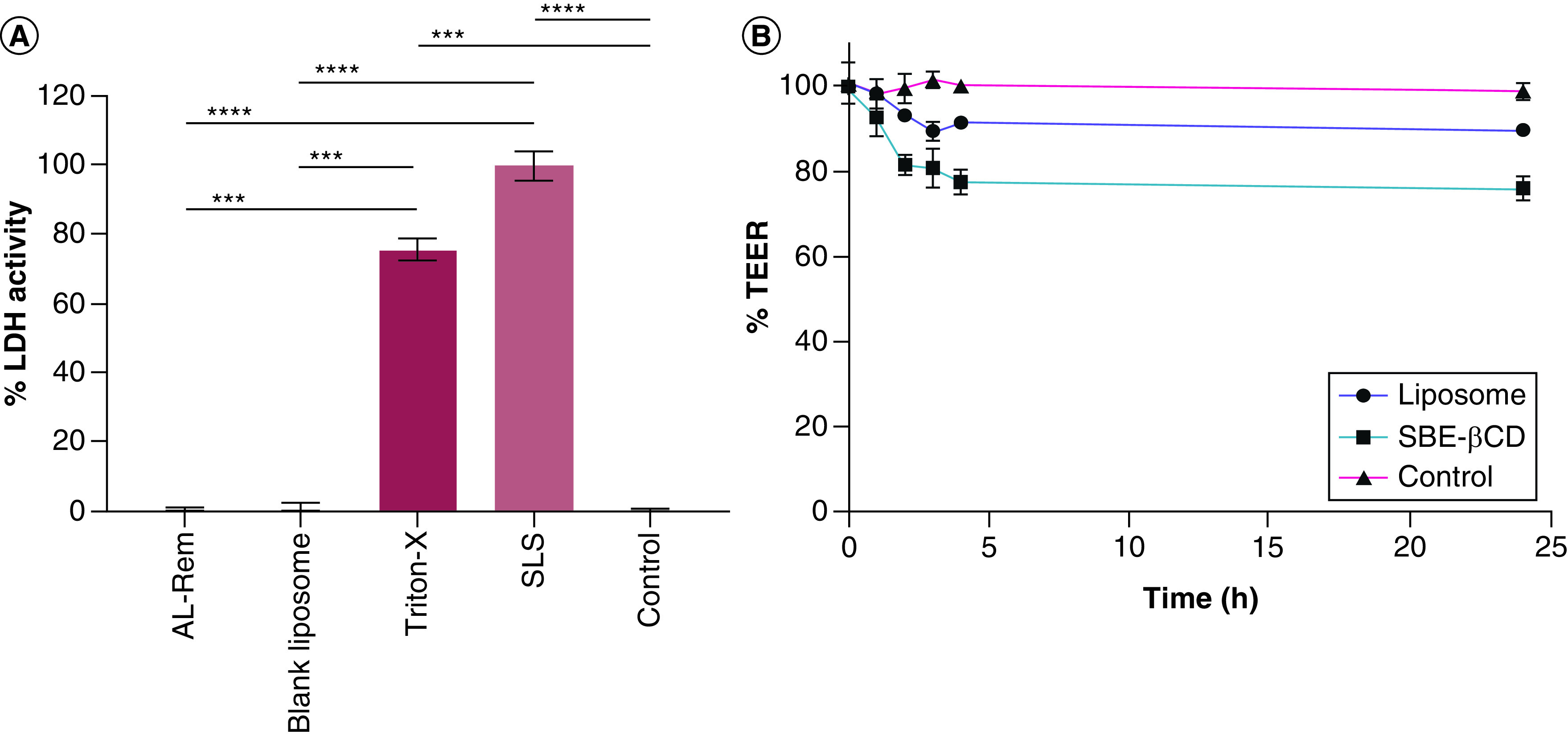
Percentage of LDH released and TEER reduction assay post-treatment. **(A)** Percentage of LDH activity in A549 cells after 3-h incubation with either AL-Rem or blank liposomes was found to be 75-fold less compared with positive control. **(B)** Transepithelial electrical resistance values of Calu-3 monolayer after 4 h showed 10 and 20% reduction post-treatment with either liposomes or SBE-β-CD (50 mg/ml). Data obtained for treatment groups were compared with the control group (no formulation). Data represent mean ± standard deviation (n = 3). ***p ≤ 0.001; ****p ≤ 0.0001. AL-Rem: Aerosolized nanoliposomal carrier for remdesivir; LDH: Lactate dehydrogenase.

Cell membrane integrity can be effectively measured by quantifying the activity of cytoplasmic enzymes released because of cellular damage. As per Figure 4A, in comparative analysis, Triton X-100 used as positive control showed a significantly higher amount of LDH release (p ≤ 0.001) compared with AL-Rem and blank liposomes, and the use of 0.1% w/v SLS showed immediate and highest membrane disruption (i.e., nearly 100%). By contrast, AL-Rem and blank liposomes, equivalent to 2.5 mg/ml Rem concentration, showed an absorbance value similar to that of the control group (i.e., cells with media), indicating no cell membrane disruption.

### Epithelial integrity study

TEER is used to measure electrical resistance across a cellular monolayer in the presence of a medium. During the 24-h incubation with liposomes, Calu-3 cells showed less than 10% reduction in TEER compared with the original value. In comparison to the negative control (i.e., cells with media), there was only a 9% reduction in TEER value at the end of 24 h. By contrast, treatment with SBE-β-CD at 50 mg/ml showed a 20% TEER reduction after 4 h. As depicted in Figure 4B, a nearly 25% reduction in TEER was observed at 24 h.

### *In vitro* release study

**Figure 5.**
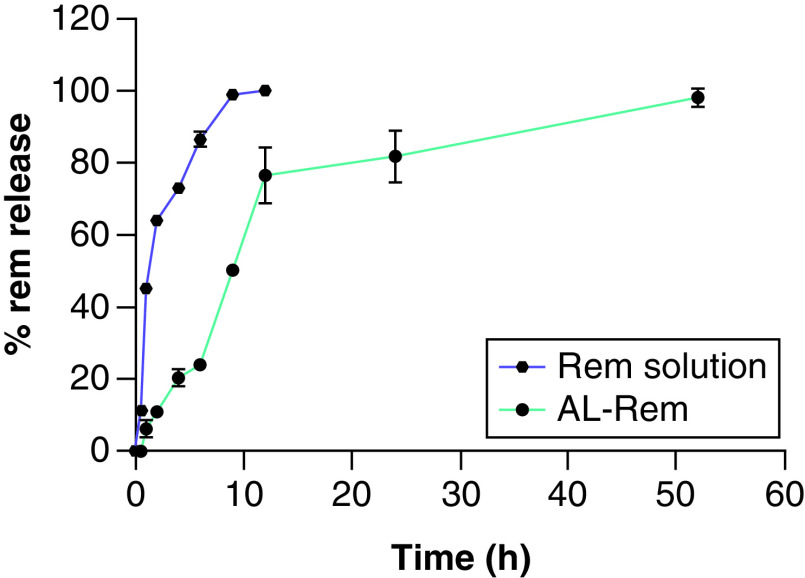
*In vitro* release in Simulated lung fluid media. Rem solution showed complete drug release within 8 h. AL-Rem sustained drug release and showed complete drug release within 50 h. Each data point represents mean ± standard deviation (n = 3). AL-Rem: Aerosolized nanoliposomal carrier for remdesivir; Rem: Remdesivir.

*In vitro* drug release of AL-Rem was evaluated using the dialysis cassette method. The release behavior of Rem from Rem solution and AL-Rem nanoliposomes is summarized based on cumulative release of Rem in SLF conditions (Figure 5). The HPLC area measurements obtained were used to calculate the corresponding concentration values. Rem solution showed very rapid and complete drug release within 8 h. By contrast, AL-Rem showed incremental drug release during the initial 10 h, later achieving a sustained release pattern. More than 75% drug release was observed after 10 h, and complete drug release (i.e., nearly 2.5 mg of Rem) occurred within 50 h.

### Liposomal stability study

**Figure 6.**
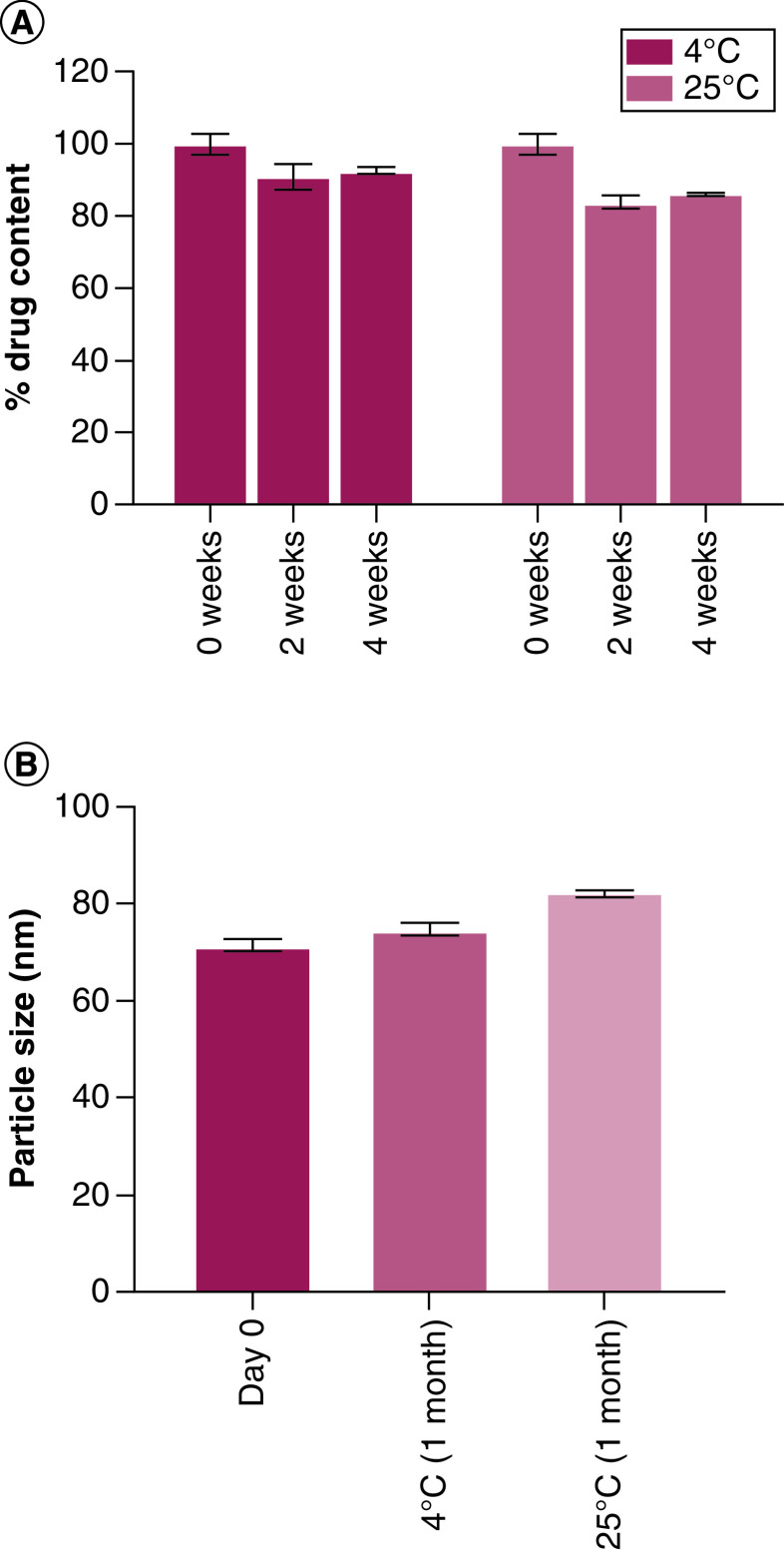
Stability analysis of aerosolized nanoliposomal carrier for remdesivir in solution. **(A)** Percentage of drug remaining after 2 and 4 weeks of storage at 4°C and 25°C. **(B)** Particle size analysis after 1-month stability study. No significant change in particle size was observed at 4°C.

AL-Rem stability was investigated for 1 month in solution form. Liposomal membrane lysis following 2 and 4 weeks of stability confirmed more than 90% drug entrapment when stored at 4°C. Additional reduction in drug entrapment was noted when stored at 25°C, with more than 15% drug loss after 2 and 4 weeks (Figure 6A). Drug loss could be due to hydrolytic degradation over time. Stability of the liposomes was further characterized by particle size analysis. As shown in Figure 6B, AL-Rem particles after 1 month of storage at 4°C showed minimal change in overall size. However, a minor increase in particle size was observed when stored at 25°C. Therefore, storage of AL-Rem liposomal preparations at 4°C is more desirable.

### *In vitro* aerosol performance & pulmonary drug deposition

**Figure 7.**
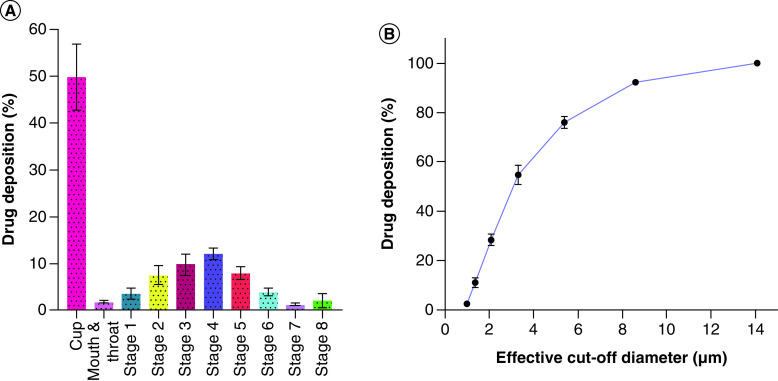
Pulmonary drug deposition profile of aerosolized nanoliposomal carrier for remdesivir. **(A)** Aerosol dispersion performance depicted as percentage of drug deposited in each stage of next-generation impactor. Each impaction stage has a set cutoff diameter as follows: stage 1 (14.1 μM), stage 2 (8.61 μM), stage 3 (5.39 μM), stage 4 (3.3 μM), stage 5 (2.08 μM), stage 6 (1.36 μM) and stage 7 (0.98 μM). **(B)** Percentage of cumulative deposition representing the percentage of particles retained in each stage.

After nebulization, the aerodynamic properties of liposomes influence the drug deposition profile (i.e., amount deposited in alveolar deep lung regions and airways). The aerodynamic particle size distribution of the aerosol, MMAD, geometric standard deviation and spread of the aerodynamic particle size distribution were estimated using a Next Generation Cascade Impactor. The MMAD of the AL-Rem was 4.56 ± 0.55 μm, with a geometric standard deviation of 2.27 ± 0.12 μm, implying that the emitted dose would be deposited in the gas exchange regions of the lungs. The FPF was found to be 74.40 ± 2.96%, suggesting good aerosolization performance of the liposomes. Aerosol deposition profile and plot of the percentage of cumulative drug deposition for each stage of NGI are presented in Figure 7A & B. These *in vitro* data illustrate that the AL-Rem formulation is suitable for inhalation delivery, with deposition deep in the lung airways.

## Discussion

An important determinant of coronavirus entry into mammalian cells is its viral infectivity and pathogenesis. It is therefore regarded as a chief target for human intervention strategies [25]. Coronavirus infection is known to be more pronounced in the lung airways [26]. Presently, patients hospitalized with COVID-19 are treated by intravenous administration of Rem. Drug distribution and formation of a polar Rem metabolite are two of the concerning factors related to the injectable formulation [9]. Moreover, the systemically administered drug must reach the lungs at therapeutic levels to exert its effect. During the pandemic, the injectable administration of Rem has been inaccessible to most patients because of limited occupancy in hospitals and professional supervision. To offer Rem to all patients who need it, more convenient and accessible dosage forms must be promptly developed. The inhalation route is a promising alternative for Rem formulation, as it facilitates direct delivery to the main site of infection. An inhaled Rem formulation would maximize drug exposure at the target site without first-pass metabolism, enhance antiviral efficacy in the lungs and minimize potential systemic toxicities [27]. Also, direct administration to the lungs would be much cheaper compared with intravenous injection owing to minimized dosing frequency and amount of drug delivered. Moreover, self-administration of inhaled Rem in patients without professional administrators would provide affordable and early-stage treatment of COVID-19 in these challenging times [28].

In the current study, preliminary evaluation involving pH- and temperature-based stability revealed the hydrolytic degradation potential of Rem. With the increase in temperature, the amount of degradation was found to substantially increase. Similarly, the drug was found to be more susceptible to degradation in alkaline pH conditions. Therefore, conventional solubility enhancement methods like pH adjustment and salt formation would result in higher chances of chemical degradation. In this case, nanocarrier-based approaches, especially liposomes, could offer the desired benefits, as they are capable of being aerosolized into particles that have the desired size range and high FPF and they can encapsulate a pharmaceutically viable concentration of the drug into lipid vesicles that exhibit prolonged release at the target site [29]. Furthermore, liquid liposomal formulations can be delivered effectively through medical air-jet nebulizers, making this an easy and preferred drug delivery design [17,30]. For example, delivery of doxorubicin-encapsulated liposomes via inhalation induced considerably higher cell death at the target tissue in mouse lungs and substantially reduced negative side effects in nontarget organs, such as kidneys, liver and spleen, when compared with free doxorubicin or intravenously administered doxorubicin-encapsulated liposomes [31]. Moreover, as only a portion of the drug gets released at a certain time, the drug encapsulated within the liposomal barrier is safe from chemical degradation, which helps increase the therapeutic efficacy of the drug.

Based on the literature, different commonly used methods for liposome preparation were screened. The most convenient approach (i.e., the ethanol injection method) showed rapid drug precipitation. This could be due to rapid dilution of ethanol in the aqueous phase and low solubility of the drug in ethanol and aqueous medium. Therefore, one of the novel techniques (i.e., the modified hydration method) was utilized. As reported previously, higher physical stability and entrapment efficiency of Rem were achieved with the modified hydration method. As per Patel *et al.*, high entrapment was possibly the result of rapid distribution of the drug within the inner hydrophobic lipid layer, thus restricting the ability of the drug to diffuse out in the aqueous phase [32]. Moreover, because of the presence of mannitol or osmotic pressure generation, more drug was favored in the inner lipid layer than the outer lipid layer during the hydration step, promoting enhanced stability [20,32]. Liposomal formulations with compositions comprising lung surfactants like DPPC could be potential carriers for pulmonary drug delivery owing to their high biodegradability, controllable surface charge, sustained release behavior and capability of encapsulating hydrophobic compounds like Rem [33,34]. However, the authors' results showed significant cytotoxicity to A549 cells with Rem-DPPC-based liposomes at the desired concentration, and the cells were also unable to hold Rem for a prolonged period. Therefore, an additional member (i.e., DOPC) of the same class of unsaturated lipids was incorporated into the formulation. However, combining the two lipids also failed to entrap the drug for a prolonged period in the aqueous environment. This can be attributed to the fact that unsaturated fatty acids help maintain membrane fluidity partially by reducing the packing between phospholipids, whereas saturated fatty acids only increase the packing between phospholipids, thus reducing membrane fluidity [35]. Additionally, because of the double bond present in DOPC, it is known to produce a less ordered bilayer with a high cross-sectional area, thus aiding drug encapsulation for a prolonged period. As shown by Leekumjorn *et al.*, increasing palmitate concentration is directly related to an increase in drug leakage from the liposomal barrier [35]. Therefore, compared with DPPC and DODP liposomes, which have a higher concentration of palmitate, DOPC showed minimal drug leakage even after 4 weeks of storage. Since Rem liposomes were prepared using a commonly used technique and phospholipids, microscopic evaluation (e.g., transmission electron microscopy) was not carried out.

Focusing more on the practical aspects, as per the study by Sun, Rem could show better efficacy *in vivo* via the pulmonary route when the projected drug dose is approximately 10.5 mg of its active metabolite (Nuc-TP) [13]. Therefore, one of the best ways to deliver a high concentration of Rem is by using an air-jet nebulizer. Aerosolizing Rem alone would involve complexities like drug hydrophobicity, rapid hydrolysis to a hydrophilic metabolite that cannot enter lung cells or instability in aqueous solution. Therefore, Rem liposomal aerosols (AL-Rem) could significantly extend the drug retention half-life, reduce the dose volume, improve intracellular diffusion and bioavailability, enhance tissue tolerance against high drug dosages and minimize pulmonary clearance by protecting the encapsulated drug from enzymatic degradation [29]. In the present study, AL-Rem was completely stable and showed more than 90% A549 cell viability up to the highest concentration tested. With 2.5 mg/ml of Rem in liposomes and 75% FPF, nebulizing a volume of 6 ml would theoretically be enough to achieve the desired lung concentration. Moreover, because of the hydrolytic degradation of Rem alone, it is suggested that the duration of nebulizer inhalation not exceed 1–2 h [13]. However, as Rem is encapsulated within the liposomal barrier, the chances of Rem undergoing hydrolytic degradation are minimal, thus widening the scope with regard to increasing the duration and volume of nebulization.

AL-Rem also offered additional benefits, which were illustrated using different assays. Physicochemical characterization revealed the hydrodynamic diameter of liposomes to be 71.46 nm, with a highly negative zeta potential. Charged particles have a better tendency to inactivate viral particles in comparison to neutral molecules. They can affect the overall integrity of the viral particle because of surface adsorption, protonation and deprotonation of surface moieties [36,37]. Reports state that negatively charged particles are slower acting than positively charged particles but more effective. Moreover, they show lesser toxicity to normal mammalian cells in comparison to positively charged particles [38]. In addition, for the drug to show efficacy, it must be released at the target site. Therefore, to provide clinically relevant information, drug release was carried out in SLF with pulmonary surfactant. AL-Rem showed sustained release of Rem compared with Rem solution. Based on the literature, Rem has shown an IC_50_ of 0.987 μM against SARS-CoV-2, which would correspond to approximately 0.6 μg/ml [39]. Since liposomal drug concentration is around 4166 times higher than the IC_50_, a release of even 10% of the drug should show a potent effect against SARS-CoV-2. Additionally, sustained release would compensate for the need for frequent dose administration.

*In vitro* cytotoxicity data confirmed the liposomes to have no significant toxicity toward mammalian lung cells. Unlike SLS, which shows rapid membrane disruption, AL-Rem showed no cellular damage, which was quantified by the amount of LDH released. Airway lung epithelial disruption is often associated with increased permeation of virions to the systemic circulation. Several autopsy studies involving histopathological observation have confirmed severe alveolar damage with necrosis of alveolar lining cells in the presence of virions [40]. In such cases, even minimal toxicities induced by a formulation can complicate the disease and allow easy permeation of virions. Epithelial integrity of Calu-3 cells gave only a 10% reduction in TEER value at the highest liposomal concentration. However, other drug stabilizers like cyclodextrin showed a comparatively higher reduction in TEER value. This could be correlated with the ability of cyclodextrins to act as permeation enhancers by reversibly opening tight junctions [41,42]. Additionally, as the average lung fluid volume is approximately 0.37 ml/kg, which would be four to five times higher in volume than the nebulized dose, the chances of any epithelial disruption would be very minimal. Apart from cytotoxicity, liposomal stability is an important parameter that directly affects the therapeutic efficacy of the encapsulated drug and is interrelated with the manufacturing steps, storage and delivery [43]. Therefore, evaluation involving long-term stability in aqueous media was carried out by sampling the liposome for particle size and percentage of drug retained after 2 and 4 weeks of preparation. The extent of liposomal instability in various biological systems, such as plasma and water, is directly dependent on the relative drug concentration, size, lamellarity, lipid composition and incubation temperature [44]. The two most important characteristics – namely, size and incubation temperature – were analyzed. Liposomes showed higher stability and no change in particle size when refrigerated at 4°C. By contrast, the amount of drug recovered post-incubation at room temperature was comparatively less, and there was an increase in particle size, which may have been due to aggregation or swelling of liposomes. Therefore, it is recommended to refrigerate the AL-Rem preparation during storage. Another approach could involve lyophilizing the liposomes and reconstituting them in clinical settings before administration.

Pulmonary drug delivery is accompanied by various barriers that limit aerosol deposition deep in the lungs (i.e., the alveolar region and respiratory bronchioles), and these are the anatomical barrier, the pathological barrier and the immunological blockade [45]. In addition, for an inhaled formulation to be deposited in the alveolar regions and be therapeutically effective, the aerodynamic particle size of the inhaled particle should be within the range of 1–5 μm with FPF. The amount of active pharmaceutical ingredients emitted from the nebulizer and FPF are key parameters that impact clinical outcomes [46]. Based on the literature, an air-jet nebulizer was used because of its higher efficiency and FPF. In the current study, MMAD and FPF percentage confirmed the respirable potential of formulated liposomes and efficient lung deposition. Usually, particles with MMADs between 1 and 5 μm facilitates entry into the deep lungs. Also, particles most likely to undergo elimination by resident alveolar macrophages are between 2000 and 3000 nm [47]. As the aerodynamic particle size range of the authors' liposomal formulation was 4.56 ± 0.55 μm, with the added benefit of PEGylation, it is more likely to circumvent the respiratory clearance pathways and reach the gas exchange areas of the lungs easily. Taken together, these results provide strong evidence that delivering Rem as an aerosolized liposomal preparation (i.e., AL-Rem) directly to the lungs would provide several added advantages, with long-term stability and reduced dose administration, in comparison to the currently marketed preparation. In the context of the aforementioned data, which present an extensive characterization of AL-Rem liposomes *in vitro*, preclinical testing confirming the efficacy of AL-Rem in a relevant animal model could be a vital future study.

## Conclusion

The current research demonstrates the successful development of a nebulized and scalable dosage form of Rem intended for self-medication to combat COVID-19. The use of FDA-approved phospholipids and modified hydration method with a commonly used particle size reduction method makes AL-Rem viable for large-scale manufacturing. The formulated nanoliposomes of Rem (AL-Rem) have optimal particle size, effective aerosol characteristics and high drug entrapment efficiency. Minimal mammalian cell toxicities and prolonged drug release will benefit local administration, reduce frequent dosing and enhance the therapeutic efficacy of the drug. Minimal loss of cell membrane integrity would combat systemic invasion of the virus. Although the preliminary results are promising, preclinical studies need to be performed to demonstrate the clinical relevance.

## Future perspective

COVID-19 represents a sudden and enduring viral outbreak that is currently being faced by humankind. In such a scenario, drug repurposing is the fastest treatment option available. The currently available therapy (i.e., Rem injection) has some major challenges. Our study demonstrates a more effective localized delivery of Rem, which has the potential to overcome the hurdles related to the injectable form. Moreover, the development of an aerosolized nanoliposomal carrier can further potentiate drug efficacy and has great translational potential. Such a carrier could also serve as a promising drug delivery system for self-administration, combating systemic toxicities and helping to enhance therapeutic levels of the drug in the lungs.

**Summary points**

- Remdesivir, an effective repurposed drug against SARS coronavirus 2, was formulated as a localized nanoliposomal drug delivery system to maximize drug deposition in the lungs and circumvent systemic toxicities.
- An aerosolized nanoliposomal carrier for remdesivir (AL-Rem) was prepared using a modified hydration technique, with successful entrapment of 99.79% of remdesivir, and resulted in a nanosized carrier with a negative surface charge and unimodal size distribution.
- AL-Rem showed negligible cytotoxicity in lung adenocarcinoma cells and minimal disruption of lung airway epithelial cells.
- AL-Rem revealed a sustained drug release pattern, with complete release achieved within 50 h in simulated lung fluid conditions, indicative of prolonged activity, with reduced frequent dosing.
- AL-Rem solution was found to be physically and chemically stable at 4°C even after a month.
- Most importantly, desirable performance after aerosolization of nanoliposomes confirmed significant deposition of remdesivir within the gas exchange areas of the lungs.
